# A serum mesothelin level is a prognostic indicator for patients with malignant mesothelioma in routine clinical practice

**DOI:** 10.1186/1471-2407-14-674

**Published:** 2014-09-17

**Authors:** Mark Linch, Spyridon Gennatas, Stanislav Kazikin, Jhangir Iqbal, Ranga Gunapala, Kathryn Priest, Joanne Severn, Alison Norton, Bee Ayite, Jaishree Bhosle, Mary O’Brien, Sanjay Popat

**Affiliations:** Royal Marsden Hospital, Fulham Road, SW3 6JJ London, Surrey UK

**Keywords:** Biomarker, Mesothelin, Prognosis, Response

## Abstract

**Background:**

Malignant mesothelioma (MM) carries a poor prognosis and response rates to palliative chemotherapy remain low. Identifying patients with MM that are unlikely to respond to chemotherapy could prevent futile treatments and improve patient quality of life. Studies have suggested that soluble mesothelin is a potential biomarker for early diagnosis and prognosis of MM. We set out to explore the utility of serum mesothelin in routine clinical practice.

**Methods:**

We conducted a prospective exploratory study of serum mesothelin levels in 53 consecutive patients with MM at our institution between April 2009 and February 2011. Survival was assessed and analysed by mesothelin level as both continuous and categorical variables using Cox regression models. Differences in response rate between treatment groups were assessed by the Kruskal-Wallis Test.

**Results:**

All 53 patients, who had been given study information agreed to participate. The patients’ median age was 69 (range 24–90). Median mesothelin level was 2.7 nM and this value was used to dichotomize categories: ≤2.7 nM (low) and >2.7 nM (high). The progression free survival (PFS) for low vs high mesothelin was 8.0 vs 5.1 months (HR 1.8, p-0.058). When mesothelin was accessed as a continuous variable for PFS the HR was 1.03 (95% CI: 1.01 - 1.06; p = 0.013). The overall survival (OS) for low vs high mesothelin was 17.2 vs 11.3 months (HR 1.9, p = 0.088). When mesothelin was assessed as a continuous variable for OS the HR was 1.02 (95% CI: 0.99 - 1.04; p = 0.073). Thirty patients received chemotherapy of which 18 had a pre-chemotherapy serum mesothelin level. In these 18 patients, the pre-chemotherapy mesothelin level did not correlate with response.

**Conclusions:**

A single random sample provides information about patient prognosis but does not predict treatment response. We suggest further prospective validation of mesothelin testing as a prognostic biomarker.

## Background

Malignant mesothelioma (MM) is an aggressive cancer of serosal surfaces such as the pleura, peritoneum and rarely the pericardium. It is causally linked to asbestos exposure with a lag time of 15–60 years and has an incidence of approximately 2500 cases/year in the UK [1]. Diagnosis of MM is challenging as symptoms and early radiographic signs are often non-specific and their significance can be masked by multiple co-morbidities of this normally older patient. Typically, histological features of MM include positive immunohistochemical staining for epithelial membrane antigen, WT1, cytokeratin 5/6 and HBME-1 [2]. Expression of several proteins detected by immunochemistry have been suggested to correlate with survival such as IL4Rα [3], c-MET [4], aquaporin1 [5], calretinin [6], and HtrA1 [7]. The optimal surgical approach is debated and includes palliative support with or without chemotherapy contingent on co-morbidities.

The identification of a robust serological biomarker for mesothelioma could have a significant impact in this disease in helping with early diagnosis, avoiding multiple invasive procedures, providing prognostic and/or predictive information and aid in treatment response assessment. The latter is particularly important, since the response evaluation criteria in sold tumours (RECIST) and the modified RECIST criteria for mesothelioma are associated with significant variability [8, 9]. Several candidates have shown promise as predictive/prognostic biomarkers such as LDH [10], C-Reactive Protein (CRP) levels (≥1 mg/dL, predicting a poorer outcome) [11], neutrophil/lymphocyte ratio [6], platelet count (>400,000/microL, predicting a poorer outcome) [12], osteopontin [13], and fibulin-3 [14]. However, the most extensively studied is mesothelin, which has been shown to potentially differentiate between mesothelioma and other conditions, both benign and malignant [2, 15–17], and also potentially correlates with response to therapy [18]. Mesothelin is a 40 kDa membrane-localised protein that along with the 31 kDa megakaryocyte potentiation factor (MPF) are cleavage products of a 69 kDa precursor protein encoded by *MSLN* on chromosome 16. Mesothelin is proposed to play a role in cell adhesion as it binds to the cell adhesion molecule Ca125 (Muc16) and forced over-expression of *MSLN* in NIH3T3 cells leads to increased adhesion to a plastic substrate. In tissue culture, mesothelin also promotes ERK dependent proliferation [19], apoptosis resistance, anoikis resistance and invasion [20]. Mesothelin may therefore be involved in cancer metastasis and its role as a potential therapeutic target is being actively pursued [21]. It is predominantly expressed in epithelioid subtype mesotheliomas, with little/no expression in sarcomatoid sub-types. MPF and mesothelin isoforms 1 and 3 can be detected as soluble proteins in plasma or serum, which may be detected using a validated commercial dual antibody ELISA platform [16]. Mesothelin level seems to correlate with MM disease bulk and can potentially predict relapse in patients who had previously resected mesothelioma [22]. Additionally, several studies have provided some evidence for an association between high mesothelin level and poorer survival [13, 15, 23]. While the absolute baseline serum mesothelin level has not been reported to predict for treatment response a number of trials have demonstrated that a fall in the mesothelin level with treatment correlates well with radiological response rate and overall survival [24, 25].

We therefore conducted this exploratory study of serum mesothelin testing in patients with MM in routine clinical practice.

## Methods

The study was designed and submitted as a Service Evaluation. As such it fell under the remit of the Royal Marsden Hospital’s Audit Committee, which approved it without the need for a separate ethics committee approval.

### Patients

We identified patients attending our Cancer Centre with a histologically confirmed diagnosis of malignant mesothelioma.

### Mesothelin assay

The serum mesothelin assay was performed in a single laboratory. Serum samples were prospectively collected in prevalent MM cases alongside clinical data, contemporaneous to on-going patient treatment and follow-up. Levels of serum mesothelin (referred to hereafter as mesothelin) were assayed with a commercial ELISA kit (Mesomark™ Fujirebio Diagnostics, Malvern PA) according to the manufacturer’s instructions. Results were expressed in nanomoles per litre (nmol/L). This commercially available kit has passed FDA (US Food and Drug Administration) quality assurance standards. All analyses were performed in a batch, blinded to clinical outcomes.

### Treatments

Patients were treated as per local standard of care. Surgery for mesothelioma (e.g. radical pleurectomy or extrapleural pneumonectomy) was not routine practice at the time of this study. Patients that received chemotherapy were offered treatment as per the standard institutional guidelines that included research protocols. At the time of this study treatment regimens included cisplatin/pemetrexed, cisplatin/bortezomib, mitomycin/vinblastine/cisplatin and cisplatin/raltitrexed. To be eligible for anti-cancer systemic therapy, patients were required to have an Eastern Cooperative Oncology Group (ECOG) performance status ≤2 (as per local policy), have adequate renal function (clearance >60 ml/min), normal haematological indices and no serious co-morbidities, as per local guidelines. Patients underwent pre-treatment physical examination and computed tomography (CT) scan of the thorax and abdomen, as per routine clinical care.

### Response

Response was evaluated radiologically. CT scans of the thorax and abdomen were performed at baseline and following every 2 cycles of chemotherapy, as per local policy. Objective radiological response was assessed according to RECIST criteria [9]. The overall response rate (ORR) was calculated as the proportion of patients achieving a complete remission (CR) or partial remission (PR).

### Statistics

For the purposes of this study the progression free survival (PFS) was calculated from date of mesothelin measurement to disease progression or death, otherwise censored at the last follow-up date. Overall survival (OS) was calculated from the date of mesothelin measurement to death, or else censored at the last follow-up date. Survival curves were generated using the Kaplan-Meier method. Survival was analysed for mesothelin as both continuous and categorical variables using Cox regression models. For the categorical assessment, the two groups were assigned as above or below and equal to the median value. The choice of median was made prior to analysis given the small number of patients and the presence of outlying measurements on both sides of the spectrum. Any differences in pre-chemotherapy mesothelin and treatment response were assessed by Kruskal-Wallis Test and Dunn’s multiple comparison test was performed. Outcomes were not assessed by chemotherapy regime given the small numbers.

## Results

### Patient characteristics

Between April 2009 and February 2011 53 patients with malignant mesothelioma underwent random mesothelin level testing. The mean age was 69 years (range 24–90 years) and over 60% of patients had an ECOG performance status of 0–1 at the time of mesothelin testing. On histological assessment, 46 (87%) patients had epitheloid MM, 1 (2%) patient had sarcomatoid MM, 5 patients (9%) had biphasic MM and the subtype was unknown in 1 patient (2%). Forty-nine (92%) patients had pleural MM and 4 (8%) patients had peritoneal mesothelioma. There was a male predominance with 36 men and 17 women (ratio 2.1:1).

Thirty out of 53 patients received chemotherapy during their management for MM and 18/30 patients had their mesothelin level tested in the month prior to starting chemotherapy. Patient characteristics are summarised in Table 1.

**Table 1:** **Patient characteristics**

Variable	Categories		Number of patients (%)*
Patients in study			53 (100)
Mesothelioma subtype	Epitheloid		46 (87)
	Sarcomatoid		1 (2)
	Biphasic		5 (9)
	Unknown		1 (2)
Sex	Male		36 (68)
	Female		17 (32)
Age	Mean		69 (range 24–90)
Performance status	0		4 (8)
	1		28 (53)
	2		6 (11)
	3		2 (4)
	Unknown		13 (25)
Chemotherapy lines including current	0		14 (26)
	1		36 (68)
	2		2 (4)
	3		1 (2)
Chemotherapy status while on the study	Given		30 (56.6)
		Mesomark prior to CT	12 (40)
		Mesomark post CT	18 (60)
	Not given		23 (43)

*Percentages rounded up to the nearest 1.0%; Mesomark, serum mesothelin ELISA test; CT, chemotherapy.

### Random mesothelin levels

**Figure 1:**
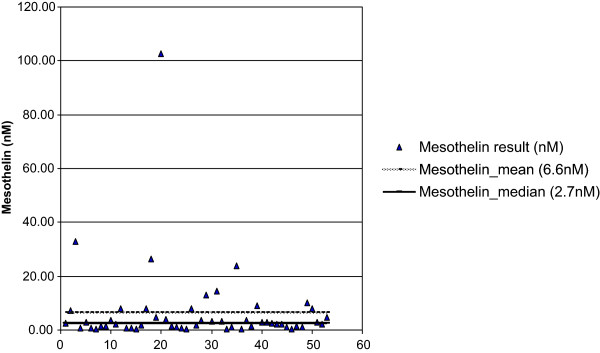
**Scatter plot of mesothelin level of study participants.**

In the 53 patients tested for serum mesothelin the mean level was 6.6 nM (range 0.3-102.5 nM). The value of 102.5 nM appeared to be an outlier and the median was calculated at 2.7 nM; the median value was chosen a priori and used for subsequent analyses (Figure 1).

### Progression Free Survival (PFS)

**Figure 2:**
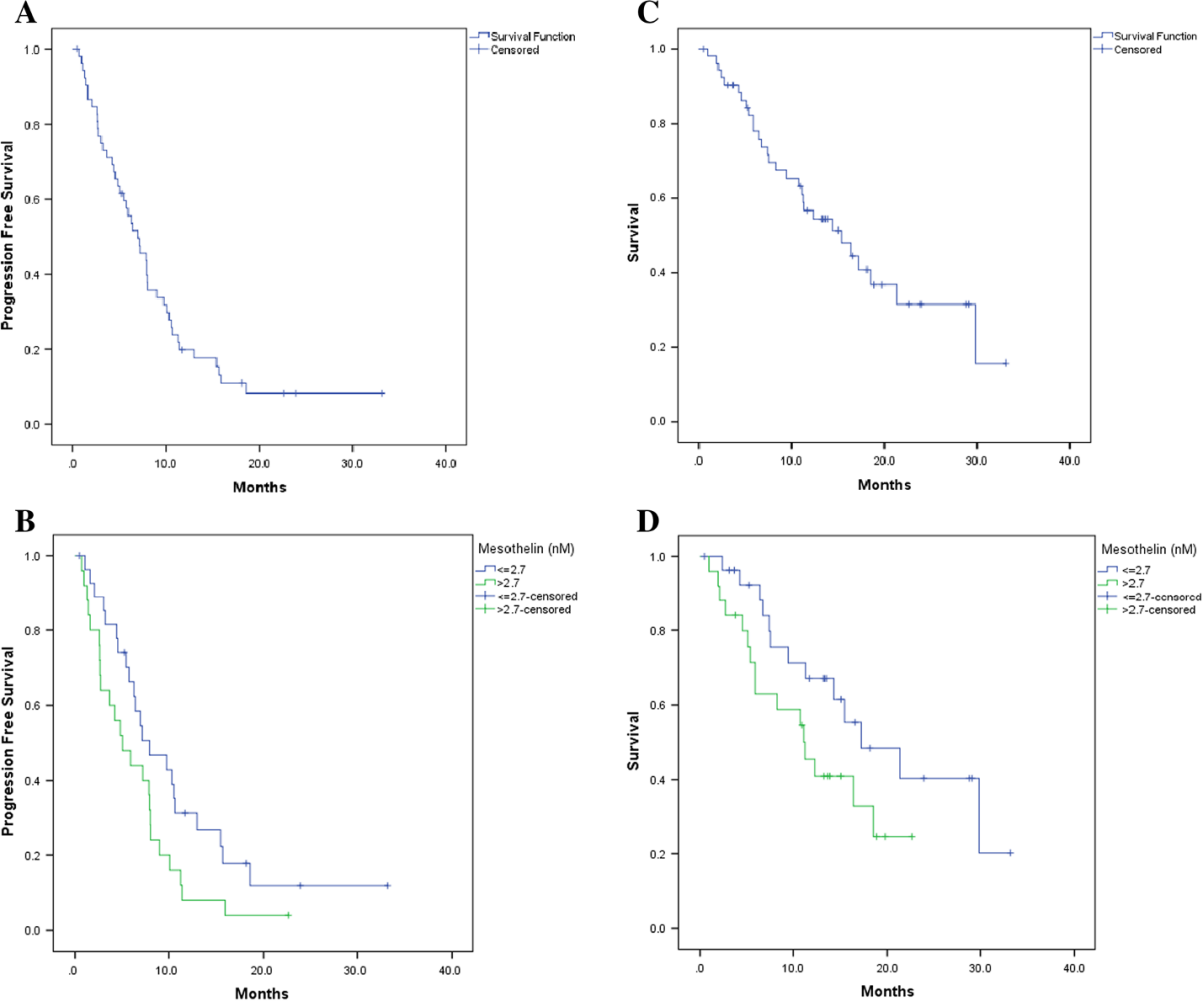
**Correlation between mesothelin level and PFS and OS. (A)** PFS with mesothelin as a continuous variable. **(B)** PFS with mesothelin as a categorical variable. **(C)** OS with mesothelin as a continuous variable. **(D)** OS with mesothelin as a categorical variable. OS, overall survival; PFS, progression free survival.

Of the 53 patients, 46 (87%) progressed and 7 (13%) were censored. When analysed as a continuous variable the Hazard Ratio (HR) for mesothelin was 1.03 (95% CI: 1.01 - 1.06; p = 0.013); for each unit increase in mesothelin the hazard of progression increased by 3%. The median PFS was 7.0 months (95% CI: 4.7 – 9.2 months) and the median follow-up for censored patients was 18.1 months (Figure 2A).When mesothelin was analysed as a categorical variable (>2.7 nM vs ≤2.7 nM) the HR was 1.8 (95% CI: 0.9 – 3.2); p = 0.059). This translated to a median of 8.0 months (95% CI: 3.9 – 12.0 months) for mesothelin levels ≤2.7 nM and a median of 5.1 months (95% CI: 2.3 - 7.8 months) for mesothelin levels >2.7 nM. The median follow-up for censored patients was 14.9 months in the ≤2.7 (nM) group and 23.8 months in the >2.7 (nM) group (Figure 2B).

### Overall Survival (OS)

Of the 53 patients assessed, 29 (55%) died and 24 (45%) were censored. When analysed as a continuous variable the Hazard Ratio (HR) for mesothelin was 1.02 (95% CI: 0.99 - 1.04; p = 0.073); for each unit-increase in mesothelin the hazard of death increased by 2%. The median survival was 15.4 months (95% CI: 9.2 – 21.6 months) and the median follow-up time for censored patients was 13.8 months (Figure 2C).When mesothelin was analysed as a categorical variable (>2.7 nM vs ≤2.7 nM) the HR was 1.9 (95% CI: 0.9 - 4.1); p = 0.088). This translated to a median OS of 17.2 months (95% CI: 8.2 - 26.2 months for mesothelin levels ≤2.7 nM and 11.3 months (95% CI: 6.7 - 15.8 months) for mesothelin levels of >2.7 nM. The median follow-up for censored patients was 13.6 months in the ≤2.7 nM group and 13.9 months in the >2.7 nM group (Figure 2D). In summary, high mesothelin levels were non-significantly associated with shorter OS when assessed as both continuous and categorical variables.

### Response rate

**Figure 3:**
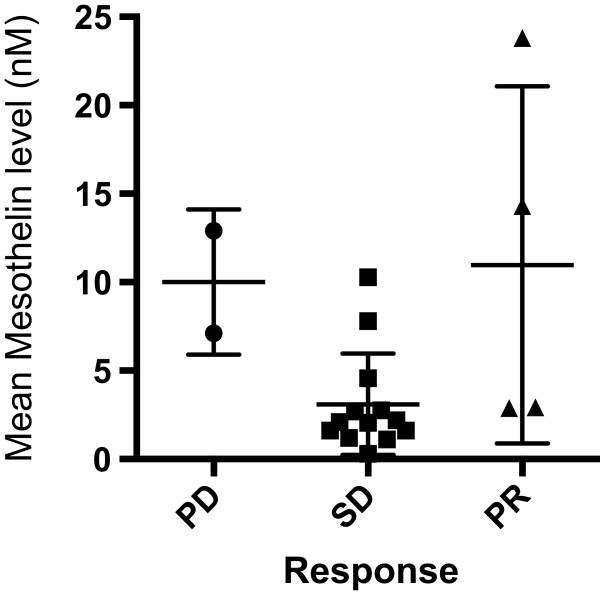
**Correlation between the pre-chemotherapy mesothelin level and treatment response attained.** PD, progressive disease; SD, stable disease; PR, partial response. The means ± standard deviation are presented. There were no significant differences between response groups.

To assess if mesothelin predicts for response to treatment, 18 patients whose sample was taken prior to chemotherapy were analysed. No patients had a complete response (CR), 5 patients (28%) had a partial response (PR), 11 patients (61%) had stable disease (SD) and 2 patients (11%) had progressive disease (PD). This gives an overall response rate (CR + PR) of 28% and a disease stabilisation rate (CR + PR + SD) of 89%. In the PD group the median mesothelin level was 10.0 (7.1-12.9), in the PR group 8.1 (2.9-23.8) and in the SD group 2.2 (0.3-10.3). Paired comparisons between the response groups found no significant differences in pre-chemotherapy mesothelin levels (Figure 3).

## Discussion

We report the use of serum mesothelin in the assessment of patients with malignant mesothelioma in routine practice. We have demonstrated an improvement in PFS in association with lower mesothelin levels when assessed as a continuous variable and a non-significant improvement in PFS with lower mesothelin levels when assessed as a categorical variable. Lower than median mesothelin levels were also associated with a better OS, which did not reach statistical significance.

The high patient accrual rate and absence of technical failures of mesothelin assessment combined with the clinically meaningful outcome measures suggest that random mesothelin is both feasible and useful for routine management of mesothelioma patients. The mean age of patients in this study was higher than our institution historical data of patients with MM treated with chemotherapy [26] and from RCT data (69 years vs 63 years) [27], although performance status was similar. Despite this older patient population, and in some cases previous lines of chemotherapy, the PFS (7.0 vs 6.1 months) and OS (15.4 vs 12.8 months) were higher than trial data for first-line chemotherapy [28]. Furthermore, it must also be noted that our definition of PFS and OS for the purposes of this study were defined from the time of mesothelin sampling to progression and/or death, respectively, and therefore potentially underestimates the true OS, PFS and differences between the studies. The improved survival in our study compared to historical controls, likely reflects a higher proportion of female patients (32% vs 20%) and epithelioid histology (87% compared to 67%), both of which are recognised to carry a better prognosis [10].

The role of mesothelin as a biomarker has been extensively studied over recent years. A number of groups have used similar techniques to demonstrate a relationship between high mesothelin levels and survival (Table 2). We have been able to confirm that these previous results are applicable to routine clinical practice and would therefore support the use of this test in this everyday clinical setting. We were unable to demonstrate any correlation between the mesothelin level and the response to chemotherapy in the 18 patients that had a pre-chemotherapy serum mesothelin level however this study was underpowered to detect such a difference. Likely to be of much greater importance is the longitudinal measurement of serum mesothelin in patients receiving treatment. A decrease in mesothelin level has already been demonstrated in patients with MM receiving cytotoxic chemotherapy [24, 25], and could become more significant still with the advent of mesothelin targeted immunotherapies. Anti-mesothelin strategies that are in early phase clinical testing include chimeric monoclonal antibodies [29], mesothelin antibody-drug conjugates [30], anti-mesothelin vaccines [31] and autologous transfer of T-cells transduced with chimeric antigen (mesothelin) receptors [32, 33]. It is possible therefore, that pre-treatment serum mesothelin levels will serve as a biomarker predictive of anti-mesothelin treatment response and that longitudinal assessment will be a measure of treatment efficacy.

**Table 2:** **Studies of mesothelin as a prognostic biomarker**

Reference	***n***	Males (%)	Median age	Receiving CT (%)	ORR (%)	OS (months)	PFS (months)
Grigoriu et al. 2007 [13]	96	81	65	85	NR	m > 3.5 = 7.0	NR
						m ≤ 3.5 = 19.0	
						P = 0.003	
Cristaudo et al. 2007 [34]	107	83.2	69	NR	NR	m > 1 = 9.8	NR
						m ≤ 1 = 21.5	
						P < 0.001	
Creaney et al. 2011 [25]	97	89	66	69		m < 1 = 20.3	NR
						m > 5 = 12.5	
						p = 0.01	
Mori et al. 2013 [35]	26	21	5	26	19.2	m < 0.469* = 26.6	NR
						m > 0.469* = 10.3	
						p = 0.027	
This study	53	68	69	57^**^	m > 2.7 = 26	m > 2.7 = 11.3	m > 2.7 = 5.1
					m ≤ 2.7 = 0	m ≤ 2.7 = 17.2	m ≤ 2.7 = 8.0
							P = 0.059^†^
						P = 0.088	

*n* = number patients; ORR, overall response rate; OS, median overall survival; PFS, mean progression free survival; NR, not reported; CT, chemotherapy; m, mesothelin level in nmol/L.

*This study used the median mesothelin index, calculated by Log2 (mesothelin level after 2 courses of chemotherapy level/mesothelin level prior to chemotherapy). Assay specifically detected N-terminal 31 kDa fragment.

**18 patients (34%) had a pre-chemotherapy mesothelin level and are therefore included in the response analysis.

^†^If the PFS is analysed as a continuous variable for every unit increase the there is a HR of 1.03, p = 0.013.

We have performed a small single institution exploratory study on the utility of serum mesothelin measurement in routine clinical practice and the findings broadly support the data from several previously published small prospective studies. Future, larger prospective studies are needed to validate the results presented here, and could be integrated with trials of mesothelin-targeted immunotherapy in mesothelioma. Additionally future studies must account for covariates, such as renal function, as subsequent to the design of our study, renal impairment was shown to lead to elevated mesothelin levels, which could reduce the accuracy of this assessment [36].

## Conclusions

In summary, our data suggests that serum mesothelin assessment is a feasible and useful test for prognostication in mesothelioma in a routine clinical setting. Single measurements of mesothelin are however of limited clinical benefit. We advocate the validation of mesothelin testing as an adjunct to chemotherapy and immunotherapies in future research protocols.

### Consent

Patient consent was not required as the study was submitted and approved as a Service Evaluation by the Royal Marsden Hospital’s Audit Committee.
